# Pembrolizumab-induced optic neuropathy – a case report

**DOI:** 10.3389/fimmu.2023.1171981

**Published:** 2023-05-09

**Authors:** Eveline Daetwyler, Alfred Zippelius, Peter Meyer, Heinz Läubli

**Affiliations:** ^1^ Division of Medical Oncology, University Hospital Basel, Basel, Switzerland; ^2^ Department of Biomedicine, University of Basel, Basel, Switzerland; ^3^ Eye Clinic, University Hospital Basel, Basel, Switzerland

**Keywords:** immune-related adverse event(s), optic neuropathy, immune checkpoint inhibitor (ICI), neuropathic, PD-1/L1, case report

## Abstract

**Background:**

Immune checkpoint inhibitor (ICI) treatment has become important for treating various cancer types, including Hodgkin’s lymphoma. However, ICI can overstimulate the immune system, leading to a broad range of immunological side effects, known as immune-related adverse events (irAEs). Here, we report a case of optic neuropathy caused by pembrolizumab.

**Case presentation:**

A patient with Hodgkin’s lymphoma received pembrolizumab every three weeks. Twelve days after the sixth cycle of pembrolizumab, the patient was admitted to the emergency department with blurred vision, visual field impairment and altered color perception affecting the right eye. The diagnosis of immune-related optic neuropathy was established. Pembrolizumab was stopped permanently and high-dose steroid treatment was immediately started. This emergency treatment led to a satisfactory binocular vision and an improvement of visual acuity testing results. After another 7 months, the left eye was affected with the same symptoms. At this time, only an extended immunosuppressive therapy consisting of high-dose steroid treatment, plasmapheresis, immunoglobulin treatment, retrobulbar injection of steroids and mycophenolate mofetil, successfully reduced the symptoms.

**Conclusions:**

This case highlights the need for prompt recognition and treatment of rare irAEs, such as optic neuropathy. Urgent treatment with initial high-dose steroid treatment is required to avoid persistent loss of visual acuity. Options for further treatment are mainly based on small case series and case reports. In our case, a retrobulbar injection of steroids in combination with mycophenolate mofetil showed significant success in treating steroid-refractory optic neuropathy.

## Introduction

Immune checkpoint inhibitor (ICI) treatment has established its relevance in refractory and relapsed Hodgkin’s lymphoma (1, 2). While being effective, ICI treatment is associated with a broad spectrum of immune-related adverse events (irAEs) (3–5). Thereby, ocular irAEs are rare side effects, but can have a major impact on the quality of life in the case of impaired vision or complete loss of vision, respectively (3–7). In this report, we present a challenging case of pembrolizumab-induced bilateral optic neuropathy. It first occurred in the right eye, which was successfully treated with high-dose steroids. Seven months later, the left eye was affected too and despite high-dose steroids, plasmapheresis and immunoglobuline treatment, no persistent clinical benefit was achieved. Only the administration of a retrobulbar injection of steroids in combination with mycophenolate mofetil led to a sustained success.

## Case presentation

A 67-year-old woman was diagnosed with Hodgkin’s lymphoma (initial disease stage according to Ann Arbor classification: IIA, without risk factors) which continued to progress after initial standard treatment with ABVD (liposomal doxorubicin, bleomycin, vinblastine, dacarbazine). Due to disease progression, two cycles of ICE (ifosfamide, carboplatin, etoposide) were applied, followed by brentuximab vedotin which had to be stopped after four months due to polyneuropathy. Stem cell transplantation was not possible due to a relevant pre-existing medical condition (congestive heart failure). Pembrolizumab was then started in this refractory situation. It was administered every three weeks with a dosage of 200mg intravenously. Two months after starting pembrolizumab a complete remission was achieved.

**Figure 1:**
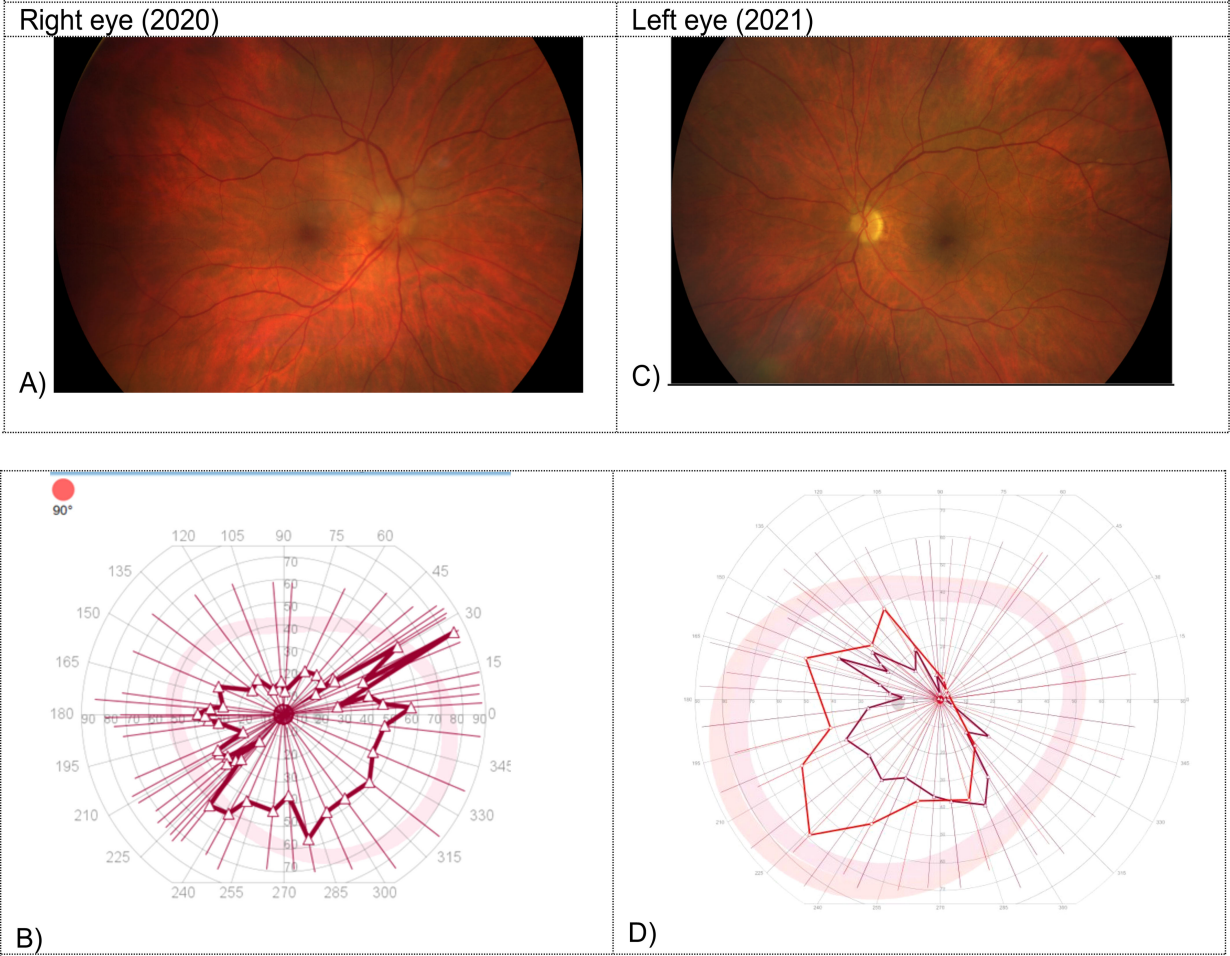
Fundus examination and visual field testing on the right in 2020 **(A, B)** and on the left side in 2021 **(C, D)**. **(A)** Papilledema in the right temporal optic disc. **(B)** Horizontal visual field loss in the visual field testing on the right side. **(C)** Sectorial papilledema in the left optic disc. **(D)** Horizontal visual field loss in the visual field testing on the left side.

Twelve days after the sixth cycle of pembrolizumab, the patient presented herself to the emergency department with blurred vision, visual field impairment and altered color perception affecting the right eye. Fundus examination showed papilledema in the right temporal optic disc (**Figure 1A**). Further testing revealed a visual acuity of 0.05 on the right side and a horizontal visual field loss (**Figure 1B**). The brain magnetic resonance imaging (MRI) showed no abnormality. The cerebrospinal fluid (CSF) analysis including extensive infectious parameters (including Borrelia species, Flavirus, Treponema pallidum, different herpes virus, measles and mumps virus), neuronal and paraneoplastic antibodies (including anti-amphiphysin antibodies, anti-Hu/anti-Yo/anti-Ri/anti-Ma/anti-Tr antibodies, anti-SOX1 antibodies, anti-ZiC4 antibodies, anti-collapsin-responsive mediator protein 5 antibodies [CRMP 5], anti-glutamic acid decarboxylase antibodies [anti-GAD], anti-angiotensin converting enzyme [ACE] antibodies), oligoclonal bands, malignant cells, flow cytometry remained without any relevant findings, despite a slightly elevated total protein level. Moreover, aquaporin-4 immunoglobulin G antibodies (ACQ4) as well as myelin oligodendrocyte glycoprotein antibodies (MOG) were not pathologically elevated. Considering these diagnostic results, the patient was diagnosed with an immune-related optic neuropathy due to PD-1 blockade with pembrolizumab. High-dose steroid treatment was immediately initiated (125mg intravenous methylprednisolone for five days), followed by tapering of prednisone over a period of four months. The symptoms showed partial regression (visual acuity of 0.3). Additional treatment was not necessarily due to a satisfactory clinical outcome regarding binocular vision. The treatment with pembrolizumab was stopped permanently. There was no sign of tumor activity.

**Figure 2:**
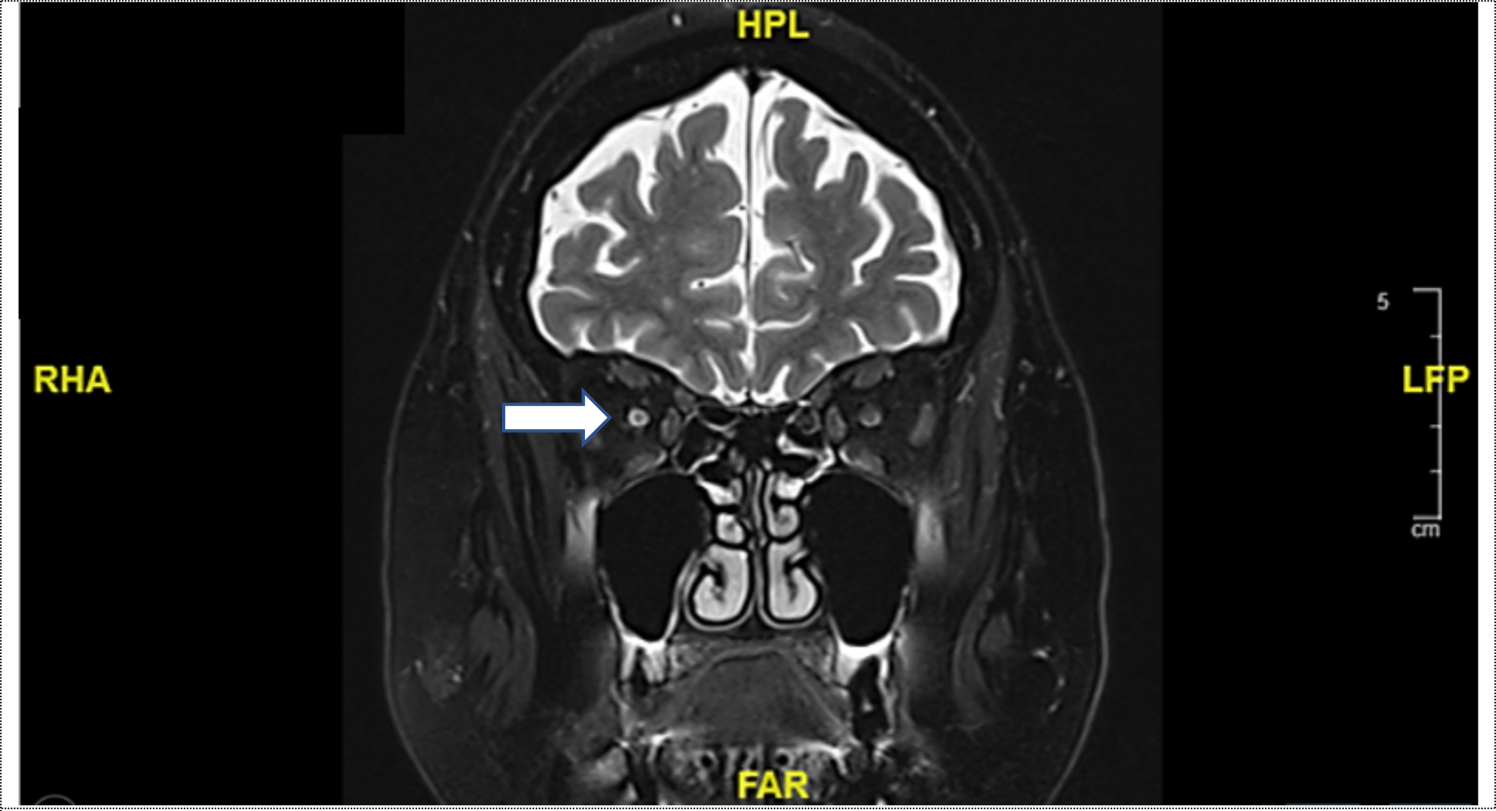
Coronal T2-weighted magnetic resonance imaging (MRI): Increasing cerebrospinal fluid (CSF) signal along the left optic nerve with continued minimal contrast uptake (white arrow).

Seven months after the initial presentation with optic neuropathy on the right side, the patient was admitted to the hospital again with complaints of blurred vision, visual field impairment and altered color perception, affecting now the left eye. Fundus examination revealed sectorial papilledema in the left optic disc (**Figure 1C**). A visual acuity of 0.4 in the left side (preliminary recording: 0.8) with horizontal visual field loss was documented (**Figure 1D**). The brain MRI revealed an increasing cerebrospinal fluid (CSF) signal along the left optic nerve with continued minimal contrast uptake (**Figure 2**). Again, an extensive examination of the CSF (same parameters as in the initial examination) showed no abnormal finding, despite a slightly elevated total protein level. In the imaging of the whole body, there was no signs of lymphoma activity. The patient was diagnosed with immune-related optic neuropathy on the left side. Treatment with high-dose steroids was immediately started (500mg intravenous methylprednisolone). In addition, plasmapheresis was performed five times after a deterioration in the symptoms as a result of a reduced high-dose steroid treatment (250mg intravenous methylprednisolone). The symptoms improved and the steroid dosage was gradually reduced. At a dosage of 80mg prednisone (1mg per kg bodyweight) the symptoms worsened again (visual acuity 0.2) and the irAE was interpreted as steroid-refractory. Immunoglobulins were intravenously administered for five days. Due to the absence of relevant clinical improvement, a retrobulbar injection of steroids was administered and treatment with mycophenolate mofetil (1000mg peroral twice daily) was started. This treatment improved the symptoms so that the steroid treatment could finally be stopped after six months of tapering. The dosage of mycophenolate mofetil could gradually be reduced (after 12 months: current dosage of 250mg peroral twice daily) without recurrence of symptoms. A visual acuity of 0.8 could be measured on the left side, corresponding to the baseline assessment. Six months after affecting the left eye the patient is independent again in everyday life. So far, there is no sign of tumor activity.

## Discussion

Ocular side effects are rare after ICI treatment (3–7). They occur in approximately 3% of patients treated with ICI, according to the FDA Adverse Event Reporting System (FAERS) pharmacovigilance database (7). It is believed that this rare occurrence is primarily due to the immune-privileged location (8). However, this can be nullified under certain conditions leading to ocular irAEs (4). Hereby, the combination of anti-CTLA-4 and PD-(L)1 is associated with a higher risk of the occurrence of these side effects compared to a monotherapy of ICI (7). The spectrum of ocular irAEs ranges from the most common ocular side effects, including ophthalmoplegia, uveitis and keratoconjunctivitis sicca (dry eye syndrome), to optic neuropathy which is much rarer (3–7, 9). In our case report, we highlight the rare complication of ICI-treatment leading to an optic neuropathy.

The clinical presentation of ICI-induced optic neuropathy is not entirely consistent with the classic triad (unilateral decreased vision, dyschromatopsia, pain), known in optic neuropathy associated with multiple sclerosis. The leading symptoms of our patient were complaints of blurred vision, visual field impairment and altered color perception which occurred with a time delay on the right, then seven months later on the left side. Pain was not reported. This is also supported by a study with 11 patients, whereas the vast majority showed painless decreased vision, floaters or both (10). Sixty-four percent of these patients showed bilateral optic neuropathy (10). Optic neuropathy occurs on average 10-20 weeks after the initiation of ICI treatment, but can also have the potential of later occurrence (6, 9, 10). The diagnosis requires observed abnormalities in optic nerve enhancement on the MRI and the clinical presentation which is consistent with the diagnosis of an optic neuropathy (3–5, 11). Therefore, prompt involvement of an ophthalmologist is mandatory (3–5). To exclude other aetiologies of optic neuropathy, such as neuromyelitis optica, autoimmune diseases, infectious or parainfectious causes, an extensive diagnostic pathway is necessary, including laboratory examinations of the blood as well as cerebral fluid analysis (3–5, 12).

After the diagnostics, the initial treatment has to be started immediately. Steroid treatment remains the backbone of this treatment, mentioned in the publications and guidelines cited below (3–6, 9, 10, 13–20) (**Table 1**). Options for further treatment are mainly based on case series and case reports. In the literature, the following additional interventions are described: plasmapheresis (n=4), intravenous immunoglobulin (n=2), infliximab (n=1), rituximab (n=1), mycophenolate mofetil (MPA) (n=1) (10, 13, 16, 20) (**Table 1**).

**Table 1:** Systemic therapeutic recommendations for ICI-induced optic neuropathy in the literature, according to the guidelines and publications (N/A = not applicable).

Systemic therapeutic recommendations for ICI-induced optic neuropathy						
guidelines, publication date author, publication date, study design with number of patients (n)	**Treatment options**					**Ocular outcome**
	steroid treatment	topical treatment	plasma-pheresis	Intra-venous immune-globulin	other immune-suppressive treatment	
ESMO guidelines, 2022 (3)	✓ systemic corticosteroids depending on severity					
SITC guidelines, 2020 (4, 21)	✓ systemic corticosteroids depending on severity					
ASCO guidelines 2021 (5)	✓ systemic corticosteroids depending on severity					
Boisseau et al., 2017, case report (n=1) (13)	✓ intravenous methyl-prednisolone 1g for 3 days, then oral steroid taper		✓ 10 sessions			n=1 with complete regression (visual acuity, MRI, optical coherence tomography)
Francis et al., 2020, case series (n=11) (13) (n=10 with treatment)	✓ oral prednisone (60mg, 80mg daily), then oral steroid taper (n=4)	✓ topical prednisolone, timolol/ dorzolamide (n=1)				n=2 with complete regression (optic nerve examination, visual field pattern)
	✓ intravenous dexamethasone 3g, then oral steroid taper (n=1)		✓ 5 sessions	✓ 1 application	✓ rituximab 3 applications	n=1 with residual defect (optic nerve examination, visual field pattern)
	✓ intravenous methyl-prednisolone 1g for 2-5 days, then oral steroid taper (n=4)	✓ topical timolol/ dorzolamide, difluprednate, brimonidine (n=1)				n=3 with residual defect (optic nerve examination, visual field pattern), n=1 with abnormal optic nerve examination (visual field pattern NA)
	✓ intravenous methyl-prednisolone 1g for 5 days, then oral steroid taper (n=1)		✓ 5 sessions			n=1 with abnormal optic nerve exa-mination (visual field pattern NA)
Kartal et al., 2018, case report (n=1) (14)	✓ intravenous corticosteroids 1g for 5 days, no taper					n=1 with improvement (visual acuity)
Kaur et al., 2019, case description (n=1) (15)	✓ high-dose corticosteroids					n=1 with improvement (symptoms)
Kim et al., 2019, case description (n=1) (16)	✓ intravenous corticosteroids			✓ (N/A)	✓ infliximab	n=1 with residual defect (optic nerve examination, visual field pattern)
Mori et al., 2018, case report (n=1) (17)	✓ intravenous methyl-prednisolone 1g for 3 days, then oral steroid taper					n=1 with residual defect (visual field pattern), but normalization of the optic nerve examination and visual acuity
Noble et al., 2019, case description (n=1) (6)	✓ intravenous high-dose corticosteroids					n=1 with improvement (visual acuity, visual field pattern)
Sengul Samanci et al., 2019, case report (n=1) (18)	✓ intravenous methyl-prednisolone 2mg/kg body weight, then oral steroid taper					n=1 with residual defect (visual field pattern, visual field acuity), but normalization of the optic nerve examination
Sun et al., 2008, case report (n=1) (19)	✓ intravenous dexamethasone, then intravenous methyl-prednisolone 250mg, then oral steroid taper					n=1 with residual defect (optic nerve examination, visual field pattern, visual acuity)
Wilson et al., 2016, case report (n=1) (20)	✓ intravenous methyl-prednisolone, then oral steroid taper		✓ 5 sessions in steroid-refractory situation		✓ myco-phenolate mofetil in steroid-refractory situation	n=1 with regression (visual field pattern, optic nerve examination) in one eye; atrophic optic nerve in the other eye
Yeh et al., 2015, case report (n=1) (22)		✓ topical prednisolone, atropine				n=1 with residual defect (optic nerve examination, visual field pattern, visual acuity)
Zhou et al., 2021, case series (n=3) (8)	✓ intravenous methyl-prednisolone					n=3 with complete regression

MPA was used in our patient due to its convincing drug characteristics, including the good tolerability and safety profile as well as its simple oral intake (23, 24). MPA reversibly inhibits the inosine monophosphate dehydrogenase which is involved in guanosine nucleotide synthesis, on which the T and B lymphocytes are exclusively dependent for proliferation (25). In addition, MPA also affects intracellular signalling pathways for lymphocyte metabolic programming (25). The efficacy of MPA is also described in optic neuropathy associated with autoimmune inflammatory disorder (23, 24). Moreover, MPA is the only drug described in steroid-refractory optic neuropathy (20). However, further investigations into the agents themselves and into the optimal sequence of these immunosuppressive interventions are required.

## Conclusion

In conclusion, we report a case with severe optic neuropathy due to PD-1 blockade showing a bilateral involvement, first of the right and then some months later of the left eye. On the right side, prompt high-dose steroid treatment showed partial success. On the left side, despite the initiation of high-dose steroid treatment at the beginning, followed by plasmapheresis and immunoglobulin treatment, the situation could only be stabilized with a retrobulbar injection of steroids and the start of treatment with mycophenolate mofetil (MPA). We emphasize the need of prompt recognition, involvement of ophthalmologists and necessity of urgent treatment to avoid substantial morbidity.

## Data availability statement

The original contributions presented in the study are included in the article/supplementary material. Further inquiries can be directed to the corresponding author.

## Ethics statement

Ethical review and approval were not required for the study on human participants in accordance with the local legislation and institutional requirements. The patients/participants provided their written informed consent to participate in this study. Written informed consent was obtained from the patient for publication of this case report and any accompanying images.

## Author contributions

ED, AZ, PM, HL participated in the care of the patient. ED drafted the manuscript. AZ, PM and HL have revised the manuscript. All authors contributed to the article and approved the submitted version.
